# Dental genetics in Brazil: Where we are

**DOI:** 10.1002/mgg3.457

**Published:** 2018-08-05

**Authors:** Priscila L. Casado, Valquiria Quinelato, Patricia Cataldo, Juliana Prazeres, Mariana Campello, Leticia L. Bonato, Telma Aguiar

**Affiliations:** ^1^ Universidade Federal Fluminense Niterói Brazil; ^2^ Universidade Federal do Rio de Janeiro Niterói Brazil; ^3^ Universidade Federal de Juiz de Fora Juiz de Fora Brazil

## Abstract

Dentistry constitutes the basic nucleus of professionals of higher level of health in Brazil with one of the largest concentrations of dentists per capita in the world. However, the genetic in dentistry in Brazil is explored, basically, in research field. Future actions need to be performed in order to deep the whole knowledge about diagnosis and treatment of diseases with genetic basis in dentistry, in Brazil.

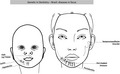

## 1 INTRODUCTION

Dentistry is the area of health that has expanded the most in recent years in Brazil, with more than 264,000 dentists. That is equivalent to almost 20% of the dentists in the entire world, according to Dentistry Federative Counsel, in 2015. Dentistry, along with medicine and nursing, constitutes the basic nucleus of professionals of higher level of health in Brazil (IPEA 2015; Morita, Haddad, & Araujo, 2010). The rate of dentists to population in Brazil is about 737 habitants per dentist. Despite that there is no consensus regarding the ideal number of dentist per capita by the World Health Organization, the general recommendation is of 1 dentist per 1,500 habitants. It means that Brazil has one of the largest concentrations of dentists per capita in the world (Saliba, Moimaz, Garbin, & Diniz, 2009). The history of dentistry and medicine in Brazil are connected, as in many other regions in the world. Professionalization of dentistry started after the expansion of sugar consumption (16th and 17th centuries) (Lucietto, 2005). In Brazil, the term “dentist” was first presented in official documents in the year of 1800 (Carvalho, 2003; Perri de Carvalho, 1995).

Formal dental education in Brazil started in 1879 with the introduction of the “Dental Surgery” discipline assigned to schools of medicine. In 1884, Dom Pedro II, the Emperor of Brazil, formalized dentistry by decree as a profession, and afterward dental courses were created in schools of medicine, in Rio de Janeiro and Bahia (Saliba et al., 2009). In 1919, dental education in Brazil developed into a 4‐year degree program that emphasized basic knowledge in biology and methodology. From 1933, dental schools become autonomous. Today, there are 220 institutions of higher education nationwide granting degrees in dentistry, with a total of about 17,157 student positions offered annually (Saliba et al., 2009).

However, Brazil still has regions with no access to oral and medicine health care. This is the main reason for the high infant mortality rate, about 18 deaths among 1,000 live births, in 2016. As a result of policies with impact on sanitation and health, this rate has progressively decreased, and as part of the Millennium Development Goals from the United Nations (UN), Brazil reached the goal of decreasing the mortality rate to two third of the one observed in 1990, 2 years before the deadline, set for 2015. In this scenario, congenital malformations have become the second‐most common cause of death in children under 1 year old, surpassing infectious diseases, and represents more than 15% of the total number of infant deaths since 2000 (Passos‐Bueno, Bertola, Horovitz, Ferraz, & Brito, 2014). These data highlighted the increasing importance of genetics knowledge for the health of the Brazilian population.

Opportunities provided by the human genome project to understand the genetic aspects of diseases and to generate novel approaches to prevent, diagnose, and manage diseases have created new imperatives for basic science and clinical education in Brazil. New knowledge has emerged in our scientific understanding of the role of genetics for diagnosing diseases and for treatments and prevention. To ensure meaningful application of genomic discoveries for preventing disease and improving clinical outcomes, the role of a professional workforce armed with leading‐edge knowledge is key to contemporary practice and education. Of equal importance is the growing evidence of the dentist's role in recognizing not only dental and oral disorders but also systemic indicators of genetic disorders, making the dentist integral to the patient's overall health and well‐being (Johnson et al., 2008).

Taking into account that not all genetic anomalies are evident at birth, dental professionals have a unique opportunity to observe the development of preadolescent and adolescent patients during periods when important growth and development changes occur. After preadolescents have completed their vaccinations (by the age of 3), they are often seen infrequently by their physician, unless specific health concerns arise. In contrast, many children are seen for routine dental care on a biannual basis regardless of their health status. Because dentists concentrate their diagnostic expertise on the face and mouth, they can be the most skilled to observe anomalies suggestive of major developmental malformations. We believe that dentists, who are able to recognize genetic disorders, can also provide a valuable service to their patients by proper referral to a medical geneticist and/or genetic counselor (Johnson et al., 2008).

In this manuscript our focus is to describe dental practice in Brazil, how genetics is applied in dentistry, how universities and researches are operating and some of our future expectations.

## 2 BRAZIL—DEMOGRAPHY AND POPULATIONAL CHARACTERISTICS

Brazil, officially the Federative Republic of Brazil, is the largest country in both South America and Latin America. At 8.5 million square kilometers (3.2 million square miles) and with an estimated population of 213.213.847 people, Brazil is the world's fifth‐largest country by area and the sixth‐most populous. The five geographic regions of the country—north, northeast, center‐west, southeast, and south—are characterized by considerable social, economic, cultural, and demographic differences. The population growth rate in Brazil was 0.77% between 2016 and 2017. From the total of 26 states, the three most populous are in the southeast region—São Paulo, Minas Gerais, and Rio de Janeiro—in contrast to the North that has a small population. The demographic projection predicts that in 24 years (between 2042 and 2043) the population will achieve its highest level (up to 228.4 million) and will decline in the following years. According to current data, more than 50% are in the age group 0–34 years, characterizing Brazil as a young country. However, this reality is changing. The fall in birth rates and the increase in the life expectancy of the population contribute to the fact that, in some decades, Brazil will be an adult country (Brazilian Institute of Geography and Statistics – IBGE, 2017).

Life expectancy at birth in Brazil increased from 75.28 years in 2015 to 75.72 years in 2017, growing at an average annual rate of 0.29% (world data atlas). The Gross Domestic Product (GDP) in Brazil was 1796.19 billion US dollars, in 2016. The GDP value of Brazil represents 2.90 percent of the world economy. GDP in Brazil averaged 634.46 USD Billion from 1960 until 2016, reaching an all‐time high of 2616.20 USD Billion in 2011 and a record low of 15.17 USD Billion in 1960 (World Bank, 2017, https://data.worldbank.org/country/brazil?view=chart), and in human development index (0.75) (http://hdr.undp.org/en/countries/profiles/BRA), reflecting an outstanding social inequality.

The Brazilian population is highly heterogeneous and admixed. Its population originated from three main ancestral roots: African, European, and Native American, the latter constituting the autochthonous population. Colonization was predominantly Portuguese (Telles, 2004). The slave trade of Africans to Brazil was the oldest, the longest running, and the largest in the Americas. Early European colonizers and their descendants brought an estimated of 3.6 million African slaves, seven times more than their counterparts in the United States (Lima‐Costa et al., 2015).

The ethnoracial classification in Brazil is more complex and fluid than in other countries, becoming difficult to correlate it with genomic ancestry. Previous genome studies based on up to a hundred informative markers showed conflicting results on this correlation (Durso et al., 2014; Ruiz‐Linares et al., 2014). The Brazilian census adopts a classification based on ethnoracial self‐classification with five groups: White, Mixed (“pardo” in official Portuguese), Black, Yellow (Asian), and Indigenous (Native American), the latter two representing <1% of the total population (Lima‐Costa et al., 2015).

A recent study exploring Brazilian mixed, based on the correlation between genome ancestry (370.539 single‐nucleotide polymosphisms) and ethnoracial classification in 5.851 community‐dwelling individuals in the South (Pelotas), Southeast (Bambui), and Northeast (Salvador) Brazil, found a significant association between the phenotype and genome ancestry, but the strength of the association varied largely across studied populations (Lima‐Costa et al., 2015). In addition, the association between Black and White self‐classification with ancestry was more consistent in the extremes of the high and low proportion of African ancestry. It was confirmed by previous historical and genetics reports of the largest African ancestry observed in Northeastern, as well as predominant European ancestry in Southeastern and Southern Brazil (Pena et al., 2011; Ruiz‐Linares et al., 2014; Santos et al., 2010).

## 3 ORAL DISEASES AS MANIFESTATION OF GENETIC DISORDERS

The largest and most comprehensive nationwide epidemiological survey on oral health ever developed in Brazil (SB Brazil 2010) was completed in 2010. Of the approximately 5,500 known inherited diseases in humans, more than 700 involve craniofacial malformations (Johnson et al., 2008). Additionally, there is mounting evidence that many complex disorders, such as diabetes and hypertension, as well as caries and periodontal disease, are the result of gene–environment interactions.

Differential diagnosis of developmental anomalies relies on the ability of the clinician to recognize and differentiate between normal and dysmorphic physical characteristics. Twelve of the 26 categories of malformations used for diagnostic purposes, according to *Smith's Recognizable Patterns of Human Malformation* (Jones, 1997)*,* involve features of the head or neck. Several are limited to oral structures such as hypodontia, microdontia, micrognathia, and cleft lip/palate (Johnson et al., 2008).

## 4 HYPODONTIA

Hypodontia (dental agenesis) is the most common developmental anomaly in humans, constituting a clinically challenging problem. Hypodontia is often used as a collective term for congenitally missing teeth, although specifically, it describes the absence of 1–6 teeth, excluding third molars (Chhabra, Goswami, & Chhabra, 2014). This tooth agenesis can either occur as an isolated condition (nonsyndromic hypodontia) or can be associated with a syndrome (syndromic hypodontia), highlighting the heterogeneity of the condition (Chhabra et al., 2014).

Hypodontia is usually associated with other oral anomalies, such as cleft lip and/or palate, reduction in size and form of teeth and alveolar processes, short root anomaly, crowding and/or malposition of other teeth, delayed formation and/or delayed eruption of other teeth, persistent deciduous teeth, impaction, anomalies of the enamel, increased free‐way space, false diastema, deep overbite, taurodontism, maxillary canine/first premolar transposition, enamel hypoplasia, and altered craniofacial growth (Camilleri, 2005; Nasman, Forsberg, & Dahllof, 1997).

Online Mendelian Inheritance in Man (OMIM; http://www.omim.org) lists over 60 different syndromic conditions that include hypodontia as part of their phenotypic spectrum of anomalies, and candidate genes have been identified for many of these conditions. Ectodermal dysplasia, oral‐facial‐digital syndromes, and syndromes with oral‐facial clefting such as Pierre‐Robin sequence and Van Der Woude syndrome are conditions associated with hypodontia. Successive linkage analysis studies have indicated involvement of different loci, mapped, respectively, to chromosome 6p24; 2p13; 19q13; and regions on 4q, in nonsyndromic cleft lip and/or palate (Chhabra et al., 2014).

### 4.1 Brazil contextualization

Although much progress has been made to identify the developmental basis of tooth formation and nonsyndromic cleft lip/palate, knowledge of the etiological basis of these inherited diseases and tooth loss is still lacking. To date, the mutation spectra of nonsyndromic forms of familial and sporadic tooth agenesis in humans have revealed defects in various genes (Chhabra et al., 2014). In this scenario, Brazil is a pioneer in genetic studies in dentistry with a high number of publications in this area, with international collaborations for studies in hypodontia. Numerous Brazilian studies on genetics in dentistry have identified the association of genetic variations in MMP9 and MMP13 (Antunes et al., 2013), FGF3 (Vieira et al., 2013), TGFB3, and BMP4 (Antunes et al., 2012), MMP1 and MMP20 (Küchler et al., 2011), MSX1 and PAX9 (Lopez et al., 2013) with tooth agenesis, in a Brazilian population.

Regarding multifactorial diseases, the three‐most common problems in dentistry, today, remain dental caries, periodontal diseases, and malocclusion. A multifactorial etiology for all three conditions has generally been assumed, with both genetic and environmental contributions to observed variability.

In complex multifactorial disease, multiple genes likely interact with environmental factors while the effect sizes attributable to individual genetic variants are likely to be small.

## 5 CARIES

Dental caries, a multifactorial complex disease (Shuler, 2001), remains the most common chronic disease of childhood. Furthermore, billions of dollars are spent for the treatment of dental caries every year and the costs keep increasing. The etiology of dental caries involves a complex interplay of environmental and genetic factors (Hunter, 1998). Epidemiological studies have tried, for many years, to understand fully the mechanisms of this disease, with the eventual goal of prevention. Thus, identifying the underlying genetic and environmental risk factors is a crucial step toward that goal.

Pioneering twin studies investigating the heritability (i.e., proportion of variation due to genes) of dental caries in children have clearly supported the key role of genetics in tooth decay (Bretz et al., 2006; Liu, Deng, Cao, & Ono, 1998). Caries heritability estimates for children based on twins range from 64% to 85%. A study by Wang et al., 2010, based on larger families, showed that heritability of dental caries in the primary dentition was over 50%. Also, they found that genes affecting caries might be different for the primary and permanent teeth. These findings have reinforced the importance of studying genetic components of dental caries in primary teeth separately (Wang et al., 2012).

Numerous efforts on gene mapping have been made so far to identify specific genetic loci contributing to caries susceptibility (Werneck et al., 2010). Results from previous studies have highlighted features involved in processes related to development of caries in the following four categories: (a) Salivary composition and flow (Stookey, 2008); (b) Tooth morphology (Guzman‐Armstrong, 2005); (c) Dietary and taste preferences (Wendell et al., 2010); and (d) Enamel and dentin formation (Wang et al., 2012). Thus, genes regulating enamel/dentin formation make significant contributions to caries development (Wang et al., 2012).

There are two plausible theories which explain how enamel genes may associate with caries lesion development: they interact with oral bacteria (such as *S. mutans*) to affect caries susceptibility (Patir et al., 2008) and/or they enhance the enamel thickness and enamel fluoride concentration to protect teeth against caries (Keene, Mellberg, & Pederson, 1980).

Besides genetics, environmental determinants also play a crucial role in caries susceptibility in children (Lukacs & Largaespada, 2006). Demographic parameters, including age, sex, and ethnicity, are important predictors for caries. In general, older children have more caries because their teeth are exposed to the environment and at risk longer; girls are typically found to exhibit higher caries prevalence rates than boys, mostly due to earlier eruption of their teeth and food preferences (Ferraro & Vieira, 2010). Moreover, caries prevalence was observed to vary among different racial groups.

### 5.1 Brazil contextualization

In Brazil, recent data from the last census, in 2010, showed 4.6% of all Brazilian children under 5 years old are free of caries. Adolescents, with mean age of 12 years old, showed absence of caries in 43.5% of total permanent dentition. From 15 to 19, 35 to 44, and 65 to 74 years old, the incidence of caries were 23.9%, 0.9%, and 0.2%, respectively (SB Brazil, 2010).

Recent studies of research institutions in Brazil tried to understand the multifactorial etiology of caries and its relationship with specific gene loci. Among numerous studies conducted in this field, major findings included:

1. Polymorphisms in nonamelogenin enamel matrix genes are associated with dental fluorosis (Küchler et al., 2018)
2. *MUC5B* gene is associated with dental caries (Cavallari, Salomão, Moysés, Moysés, & Werneck, 2018)
3. Genes involved in the enamel development are associated with calcium and phosphorus level in saliva (Küchler et al., 2017)
4. Polymorphisms in *DEFB1* and *miRNA202* are involved in salivary human β‐defensin 1 levels and caries experience in children (Lips et al., 2017)
5. A polymorphism in the *MTRR* gene is associated with early childhood caries and underweight (Antunes et al., 2017)
6. Aquaporin 5 interacts with fluoride and possibly protects against caries (Anjomshoaa et al., 2015)
7. *MMP2*,* MMP3*,* MMP9*,* MMP20*,* TIMP1*, and *TIMP2* polymorphisms are associated with white spot lesions and early childhood caries (Antunes et al., 2016)
8. Genetic influences on dental enamel that impact caries differ between the primary and permanent dentitions (Bayram et al., 2015)
9. *BMP2* gene modification is associated with caries experience in primary teeth (Romanos et al., 2015)

## 6 PERIODONTITIS AND PERI‐IMPLANT DISEASES

Periodontitis is a chronic inflammatory disease of the supporting tissues around the teeth, which results in irreversible periodontal attachment loss, alveolar bone destruction, subsequent tooth mobility, and, ultimately, if left untreated, tooth exfoliation. There are two main types of periodontitis: aggressive periodontitis and chronic periodontitis (Armitage, 1999). Severe periodontitis occurs in approximately 8%–15% of the population (Albandar & Rams, 2002; Demmer & Papapanou, 2010) depending on the definitions used for severe periodontitis and depending on the specific study population subjected to epidemiologic studies (Loos, Papantonopoulos, & Jepsen, 2015).

The complex pathogenesis of this multifactorial disease implies the involvement of a susceptible host and a bacterial challenge. Many studies have provided a valuable contribution to understanding the genetic basis of periodontal disease, but the specific candidate genes of susceptibility are still unknown. In fact, genome‐wide studies and screening of single‐nucleotide polymorphisms have yielded new genetic information without a definitive solution for the management of the diseases (Taba, Souza, & Mariguela, 2012).

### 6.1 Brazil contextualization

In Brazil, 62.9% of the children until 12 years old have periodontal health, while 50.9% of adolescents (15–19 years old) show periodontal health. This incidence decreases according to the age. In individuals from 35 to 44 years old, the incidence of periodontitis is about 67.7%, and 90% in the elderly population (SB Brazil, 2010).

As with caries, genetic susceptibility to periodontal diseases is one of the most multifactorial diseases studied in dentistry, in research community in Brazil, with numerous representative publications developed by local institutions, with Brazilian volunteers’ samples or in partnership with other countries. The main recent findings exploring the genetic bases of periodontal diseases in Brazil showed that:

1. There is relationship between suppressor of cytokine signaling 1 expression, during LPS‐induced inflammation, and bone loss in rats (Souza et al., 2017).
2. *CCR5Δ32* (rs333) polymorphism is associated with decreased risk of chronic and aggressive periodontitis (Cavalla et al., 2018).
3. Patients carrying haplotypes associated with susceptibility to chronic periodontitis can modify absolute quantification of *Aggregatibacter actinomycetemcomitans* (Cirelli et al., 2017).
4. Genome‐wide association scan for chronic periodontitis implicates novel locus for periodontitis covariants (Feng et al., 2014).

With the advent of dental implants, it has been observed that the increased failure rate in implant dentistry is associated with the development of peri‐implant disease (PID) (Lindhe & Meyle, 2008). The main causes of PID have been attributed to biomechanical failure resulting from bacterial infection and occlusal overload, in which the main risk factors are poor oral hygiene, smoking, diabetes mellitus, as well as periodontitis. Recent studies have focused on the individual genetic pattern to explain the common etiology of periodontitis and peri‐implantitis, but have yielded conflicting results and limited analyses of specific regions in a single gene (Casado, Villas‐Boas, de Mello, Duarte, & Granjeiro, 2013a; Casado, Villas‐Boas, Mello, Duarte, & Granjeiro, 2013b). However, many studies conducted in the Brazilian population, through biomolecular analysis of saliva samples, have explored the genetic bases of periodontitis and its association with PID. In the last 5 years the main publications exploring PID associated with genetic factors found that:

1. There is high level of RANKL/OPG in peri‐implant mucosae of patients with history of chronic periodontitis (Costa et al., 2018).
2. Haplotypes in *BMP4* and *FGF* genes increase the risk of peri‐implantitis (Coelho et al., 2016)
3. *MMP13*,* TIMP2,* and *TGFB3* gene polymorphisms are associated with Brazilian chronic periodontitis and peri‐implantitis (Junior et al., 2016).
4. *BRINP3* gene has different contribution in chronic periodontitis and peri‐implantitis (Casado et al., 2015).
5. *IL6* gene polymorphism is associated with periodontitis and peri‐implantitis in a Brazilian population (Casado et al., 2013a,b).

However, many multifactorial diseases affecting the orofacial region are not associated with bacterial challenge, but with other environmental factors that when associated with genetic individual predisposition can trigger disease development. This is observed in temporomandibular disorder and nonsyndromic cleft lip/palate.

## 7 TEMPOROMANDIBULAR DISORDER (TMD)

Temporomandibular disorders (TMD) are characterized by painful and/or dysfunctional changes involving the masticatory muscles and temporomandibular joints (TMJ) (Bender, 2014), and collectively, they are considered the most common cause of chronic pain in the orofacial region. According to the National Institute of Dental and Craniofacial Research (NIDCR), 5%–12% of the US population suffer or have suffered from problems related to TMD (Bonato, Quinelato, Borojevic, et al., 2017; Bonato, Quinelato, De Felipe Cordeiro, et al., 2017).

Temporomandibular disorders are heterogeneous in presentation and multifactorial in etiology (Smith et al., 2013). However, it has been hypothesized that persistent TMD pain conditions result from a “central sensitization syndrome” (Lorduy, Liegey‐Dougall, Haggard, Sanders, & Gatchel, 2013) disregarding other important etiological factors, such as the overlap of physical symptoms of TMD with those of other comorbid disorders (Morell, Camprub‐Robles, Culler, Lecea, & Delgado, 2014; Yunus, 2008). The facial pain may radiate to surrounding areas triggering jaw pain, earache, tinnitus, headache, cervical/shoulder pain, neuralgia, and toothache (Bonato et al., 2016).

However, until recently few studies have explored the genetic basis of isolated TMD and TMD with comorbid symptoms.

### 7.1 Brazil contextualization

In the field of TMD research, Brazilian research groups have been pioneers in genetic studies, in partnership with Universities in other countries. Some of the main studies found:

1. An association between polymorphisms in the genes of estrogen receptors and the presence of temporomandibular disorders and chronic arthralgia (Quinelato et al., 2018).
2. Association between Haplotypes of the *RANK* and *OPG* genes with chronic arthralgia in individuals with and without temporomandibular disorders (Bonato, Quinelato, Borojevic, et al., 2017; Bonato, Quinelato, De Felipe Cordeiro, et al., 2017).
3. Association between *ESRRB* polymorphisms with comorbidity of temporomandibular disorders and rotator cuff disease (Bonato et al., 2016).

## 8 CLEFT LIP/PALATE

Cleft lip with or without cleft palate is a common congenital disorder resulting from the lack of merging between the embryonic structures that precede the formation of lip and palate (Silva, Balderrama, Wobeto, Werneck, & Azevedo‐Alanis, 2017). About 70% of oral clefts occur as a nonsyndromic form, and the remaining 30% are associated with Mendelian disorders or chromosomal, teratogenic, and sporadic conditions. Cleft lip/palate etiology is related to a complex interplay among environmental exposures, genetic, and epigenetic factors (Machado et al., 2016; Sharp et al., 2017).

Clinically, cleft lip/palate compromises oral health, leading to missing or malformed teeth, and hampering oral hygiene. Moreover, the relationship between dental loss and oral clefts is still being studied, indicating that critical factors in the pathogenesis of the cleft lip are also critical for the odontogenesis, thus showing possible different subphenotypes (Asllanaj et al., 2017). Apart from anatomic damages, cleft lip/palate also culminates in an impact on the routine quality of life with social privation and psychological embarrassment (Ward, 2014).

### 8.1 Brazil contextualization

**Figure 1:**
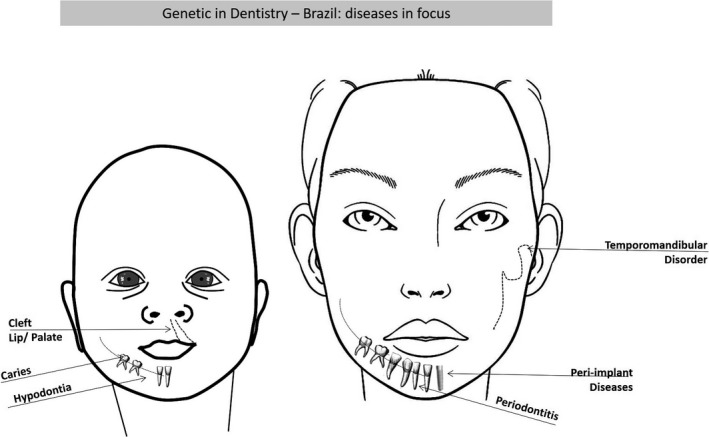
Main diseases, with genetic basis in dentistry, studied by researchers in Brazil

The prevalence of nonsyndromic cleft lip with or without cleft palate (NSCL ± P) varies according to ethnicity and geographic position; in Brazil, it is estimated at 0.99 per 1,000 live births (Panamonta, Pradubwong, Panamonta, & Chowchuen, 2015). Therefore, many researches have focused on the study of this disease and its genetic association. Figure 1 shows the main diseases explored by recent research in Brazil.

Recent studies involving Brazilian institutions and research showed that:

1. Rare variants leading to haploinsufficiency of *ARHGAP29* represent an important etiological clefting mechanism, and genetic testing for this gene might be taken into consideration in genetic counseling of familial cases (Savastano et al., 2017).
2. The high genomic instability in children with oral clefts suggests that misrepaired double‐strand breaks in DNA that create micronuclei represent a significant factor in NSCL/P development (Xavier et al., 2017).
3. *CRISPLD2* rs4783099 may represent a risk factor for NSCPO while *JARID2* rs2237138 shows a protective effect against NSCL ± P in the Brazilian population (Messetti et al., 2017).
4. Several promising candidates including *TANC2*, an oncogene required for development, and *DCAF7*, a scaffolding protein required for craniofacial development and its possibly associated with cleft development (Leslie et al., 2016).
5. The 8q24 region plays a role in CL/P and the *IRF6* G/A haplotype (rs2235371/rs642961) increases the risk for oral cleft in the Brazilian population (de Souza et al., 2016).
6. Reduced folic acid levels, alcohol consumption, and the *MTHFR* 677T and 1298C alleles may have contributed to NSCLP development in individuals from Brazil (Bezerra et al., 2015)
7. Cellular defenses against DNA damage may take part in determining the susceptibility to NSCL/P (Kobayashi et al., 2013).
8. Potential role for *ABCA4* (rs560426 and rs481931) in the etiology of CL/P in individuals from Brazil (Fontoura, Silva, Granjeiro, & Letra, 2012).

**Figure 2:**
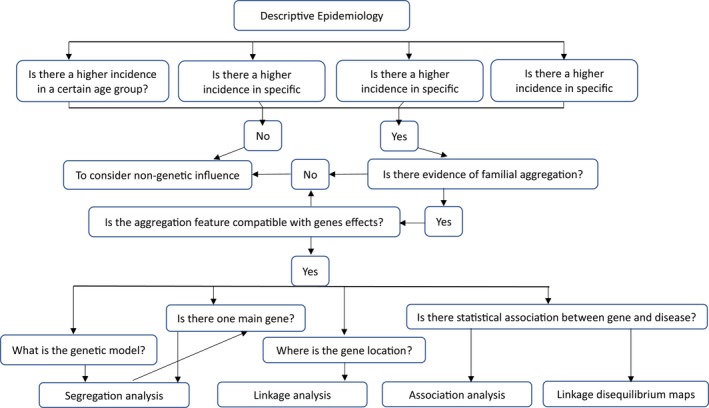
The approach to a particular problem should begin with a descriptive epidemiology of that characteristic. At this stage, variations in geographic origin, social class, age, and gender may provide clues to the involvement of genetic and/or environmental factors in the development of the phenotype. Additional data are extremely relevant as it can indicate an inheritance associated with gender

Taking into account the research conducted in Brazil that studies multifactorial diseases and their association with genetic bases and other diseases, most of the studies follow the logical sequence for investigations as a way to find the correlation of complex diseases, phenotypes, and related genes (Figure 2).

## 9 DENTAL HEALTH SYSTEM IN BRAZIL: GENETIC APPLICATIONS?

The Brazilian health system, known as the Unified Health System (SUS), follows the universal health coverage model (SUS Brazil, 2007). SUS was created by the Brazilian Federal Constitution in 1988 and it is organized by the Ministry of Health, with subsystems in each Brazilian State and city. SUS possibly represents the largest public health system in the world, and includes most aspects of health care, from outpatient care to organ transplantation, and proposes guidelines to ensure full, universal, and free‐of‐charge medical access for the entire Brazilian population. In addition to offering appointments, medical exams, and hospitalizations, SUS also promotes immunization campaigns, prevention, health monitoring, such as food control and medicament registration, and oral treatment services and campaigns (Passos‐Bueno et al., 2014).

The access to oral health services is offered by the government to all Brazilian citizens without extra (out‐of‐pocket) costs. The implementation of SUS reinforces the importance of the entire population's basic right to health access, characterizing the principle of universality. In 2004, the Brasil Sorridente Program, a social program of the Federal Government, which aims to increase the access of the needy population to the public oral treatment networks through many initiatives such as oral health education and the distribution of oral hygiene kits, was implemented.

Among the program approaches, we highlight the actions of promotion and prevention, with feasibility of fluoridation of the public supplied water; the reorganization of Primary Care in oral health, mainly with the implementation of Oral Health Teams in the Family Health Strategy; the expansion and qualification of Specialized Care, especially with the implementation of Dental Specialties Centers (CEOs); and prosthetic rehabilitation, through the Regional Dental Prosthesis Laboratories.

Before the Brasil Sorridente Program, the country had no public health policy for this area. Only those who could pay for an appointment had access to dental care in Brazil. The lack of dental care was confirmed by the SB Brazil survey carried out in 2003 by the Ministry of Health: 20% of the population had already lost all teeth, 13% of adolescents had never been to the dentist, and 45% did not have regular access to a toothbrush.

However, this scenario has been changing. According to the World Health Organization (WHO), Brazil changed this unfortunate scenario of 2003, for a position in a select group of countries considered to have low prevalence of caries. Today, there are 23.000 oral health teams throughout the country and 90% of the municipalities have at least one working team. However, before the Brasil Sorridente Program, rehabilitation in oral health was practically not effective. In 2012 alone, 410,000 total prostheses were delivered and in 2013 there were 500,000 beneficiaries.

**Figure 3:**
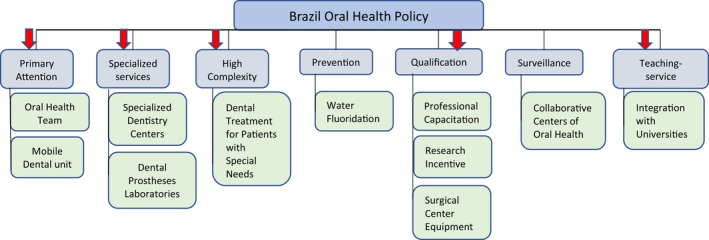
Organization of the Oral Health Policy in Brazil. However, there is a lack of correlation between genetic and clinical application. Red arrows show the steps where genetics in dentistry could be applied actually, by collecting samples for studies, applying innovations in diagnosis, or registering the incidence of the diseases

Despite the progress of the National Oral Health Policy in recent years (Figure 3), inequalities in access to dental services are still very large. There is a greater concentration of dental surgeons in the south, southeast, and mid‐west regions of Brazil. In the north and northeast regions there are approximately 1.495 inhabitants per dental surgeon. In addition, about 1.665 in 10,000 inhabitants in those regions never had a dental appointment (Correa, 2013).

Following the same feature of centralization of public health services in South and Southeast of Brazil, the panorama of our 50 years of experience in human and medical genetics shows a heterogeneous setting up of public and private genetic services across the country, with better infrastructure and development in these regions, when compared to other regions of the country (Passos‐Bueno et al., 2014). However, genetics services in dentistry are still scarce or nonexistent along some parts of the country, and it remains unclear if there are genetic services available in dentistry area, not only for treatment but also for diagnosis.

Despite a trend toward an increase in the number of dentistry courses, especially in private schools (Brazilian Federal Council of Dentistry 2017; Neto, 2002), there is a lack of systematic planning for the allocation of the dental workforce and a disconsideration of regional needs in the development of dental training programs, in Brazil, today. The adequacy of the dental workforce may be insufficient, given that there are unmet needs of oral health in low‐income populations, gaps in services coverage, and the geographic barriers (Fonseca et al., 2016).

Currently, dental schools could provide a solution to the problem of uneven distribution by addressing professional training with respect to the most relevant problems of society, including the epidemiological characteristics of regional populations. The Brazilian teaching model in practice nowadays was established by the Law of Guidelines and Bases of 1996 and the Guidelines for National Curriculum of February 2002 (The guidelines for national curriculum of dental education) which have the primary goal of preparing future professionals technically, scientifically, ethically, socially, and intellectually for the dental profession. The profile of the Brazilian professional is characterized by “generalist training and a humanist and reflexive perspective based on technical and scientific rigor, with the aim of providing service at all levels of health care.” (Law 9394, 1996).

Brazil is second in the world regarding the number of annual scientific publications in the area of dentistry. The country lags behind only the United States, and is increasing its position among the most important international scientific publications. The information was confirmed during the 34th annual meeting of SBPqO (Brazilian Society of Dental Research)—Brazilian division of IADR (Association for Dental Research)—, held in the second half of 2017 in the city of Campinas, in São Paulo. This fact deserves wide recognition. The work developed by Brazilian researchers and scientists has a practical result of extreme importance: the population benefit. That is, intense scientific production in quantity and quality means advances in methods, techniques, and equipment for patient care.

Dental research in the field of genetics has been introduced in universities, being a vital part in the development of a strong and prosperous education system in its three pillars—Education, Research, and Extension—and the research grows as the new schools of Dentistry incorporate and mature a solid research profile in their educational programs.

At the end of the 1990s, genetic engineering has been applied in dentistry, mainly in the diagnoses prediction, the modification of microorganisms that cause dental caries, and to create dental elements from stem cells, among other applications.

In this context, the EPIGEN‐Brasil Initiative is so far the largest Latin American initiative in population genomics and genetic epidemiology. Its main goal is to study the association between genetic variants found in the Brazilian population and complex diseases, taking into account one of the most important characteristics of this population: its admixture. Population genetics include genome‐wide genotyping of 6,487 individuals and high‐resolution whole‐genome sequencing from 30 individuals from three population‐based Brazilian cohorts: Salvador, Bambuí, and Pelotas.

However, the paucity of evidence of any clear‐cut single‐gene effects has meant that genetic research in dentistry has had little impact up to now on clinical dental practice in Brazil.

## 10 CONCLUSIONS AND THOUGHTS

In this manuscript, we focus on genetics in dentistry in Brazil and how universities and studies in the country are dealing with this issue, as it has direct impact on the quality of life of populations, with multifactorial or Mendelian genetic disorders involving oral health. We show that, in Brazil, research has improved in the study of genetics of oral diseases, but without clinical application on diagnosis and treatment procedures. There is no actual impact of genetic in population life, in dentistry, in Brazil.

Another relevant issue is the contrast between the part of the population that has no access to oral treatment and those who have access to private care. Therefore, in order to promote equality in health system, the strategies should include offering more jobs in the public sector, the improvement of SUS, the restructuring of the National Policy of Oral Health, and the creation of Oral Health Specialized Centers.

In addition, despite the high quality and quantity of research conducted in Brazil in genetic dentistry, dentists are not prepared, as the graduation course and the university fail to consider genetics in their clinical practice.

Dental practitioners are not well aware of the environmental and behavioral risk factors that contribute to poor oral health. Despite their routine counsel of patients about the risks of tobacco and alcohol usage, poor diet, and traumatic injuries to the head and mouth among other factors, information about the genetic makeup of individuals is still unclear; additional genetic susceptibility or resistance factors need to be applied along the dentistry community in Brazil and the dissemination of research findings needs to be discussed among research groups.

In one hand we have the state‐of‐the‐art in dentistry and we are internationally recognized, on the other, we must admit that national statistics on access to dental treatments in Brazil needs to be improved. This contradictory equation must be solved. Advances in dentistry should extend beyond the scientific area, considering genetics as the basis for the knowledge of numerous diseases that affect the population, seeking new possibilities for treatment, and reaching the private practice and public care, providing better conditions for dentists, so that there will be greater appreciation of the profession and more quality of health for the population. We believe that the realization of such a scenario depends on a broad dialogue and the union of efforts between the public power in its diverse spheres, the private initiative, the representative entities of dentistry, and the dentists as a whole.

## CONFLICT OF INTEREST

None declared.
